# A role for age-associated alterations in esophageal epithelium in eosinophilic esophagitis-associated fibrosis

**DOI:** 10.3389/falgy.2022.983412

**Published:** 2022-12-15

**Authors:** Alena Klochkova, Annie D. Fuller, Riley Miller, Adam L. Karami, Surali R. Panchani, Shruthi Natarajan, Anbin Mu, Jazmyne L. Jackson, Andres J. Klein-Szanto, Amanda B. Muir, Kelly A. Whelan

**Affiliations:** ^1^ Fels Cancer Institute for Personalized Medicine, Temple University Lewis Katz School of Medicine, Philadelphia, PA, United States; ^2^ Histopathology Facility, Fox Chase Cancer Center, Philadelphia, PA, United States; ^3^ Division of Gastroenterology, Hepatology, and Nutrition, Department of Pediatrics, Children's Hospital of Philadelphia, Philadelphia, PA, United States; ^4^ Department of Cancer and Cellular Biology, Temple University Lewis Katz School of Medicine, Philadelphia, PA, United States

**Keywords:** eosinophilic esophagitis, esophageal epithelium, aging, fibrosis, tissue remodeling

## Abstract

Subepithelial fibrosis occurs in a subset of eosinophilic esophagitis (EoE) patients and is associated with esophageal stricture. While mechanisms driving EoE fibrosis remain incompletely understood, findings from experimental systems support roles for epithelial-fibroblast crosstalk in this type of tissue remodeling. The current paradigm presents EoE as a progressive fibrostenotic disease in which aged patients develop fibrosis as a function of disease chronicity. In the current study we provide evidence that altered epithelial biology in the aging esophagus may also contribute to EoE-associated fibrosis. We find that induction of EoE inflammation in young and aged mice using the MC903/Ovalbumin protocol for the same time period results in increased lamina propria thickness uniquely in aged animals. Additionally, epithelial cells from aged mice less efficiently limit fibroblast contractility in collagen plug contraction assays compared to those from their young counterparts. Finally, to identify potential mechanisms through which aged esophageal epithelial cells may stimulate fibrotic remodeling, we perform cytokine array experiments in young and aged mice. These studies are significant as identification of age-associated factors that contribute to fibrotic remodeling may aid in the design of strategies toward early detection, prevention, and therapy of fibrostenotic EoE.

## Introduction

Eosinophilic Esophagitis (EoE) is a chronic immune-mediated, food allergen-induced inflammatory disorder characterized by the presence of eosinophil-rich infiltrates in esophageal mucosa and symptoms of esophageal dysfunction, including dysphagia and food impaction (1). In addition to the presence of inflammation, esophageal mucosa of EoE patients features evidence of tissue remodeling (2, 3), including reactive epithelial changes and subepithelial fibrosis. Fibrosis is characterized by excess deposition of extracellular matrix components in the lamina propria and is associated with esophageal stricture, the most severe consequence of EoE. Although dietary elimination and corticosteroid therapies have been demonstrated to limit progression of fibrosis in a subset of EoE patients (4, 5), therapy resistance is apparent in those with advanced fibrostenosis (6). Understanding the molecular mechanisms contributing to fibrosis in EoE has potential to guide development of approaches for early detection, prevention, and therapy of fibrostenotic EoE in order to significantly improve patient outcomes in this emerging disease.

Fibrosis involves transdifferentiation of fibroblasts in the lamina propria into activated myofibroblasts that then secrete and remodel extracellular matrix components. Transforming growth factor (TGF)-β promotes activation of esophageal fibroblasts *in vitro* (7, 8) and has also been shown to be critical for fibrosis in a murine model of EoE (9). Conditioned media from esophageal epithelial cultures has also been demonstrated to stimulate esophageal fibroblast activation and production of the profibrotic cytokines tumor necrosis factor (TNF)-α and Interleukin (IL)-1β (10). TNF-α, IL-1β, and TGF-β in the EoE microenvironment further contribute to EoE fibrosis by inducing epithelial-mesenchymal transition (EMT) in esophageal epithelial cells (10–12). As a result of this dedifferentiation process, esophageal epithelial cells gain functional characteristics associated with activated myofibroblasts, including collagen production, enhanced migration capacity, and contractility (13), allowing them to directly contribute to EoE fibrosis. These studies support epithelial-stromal crosstalk as a critical mediator of fibrotic remodeling in EoE. As epithelial-stromal crosstalk has been implicated more broadly in allergic inflammation, including studies demonstrating epithelial polarization mediated by stromal IL-4 and Interferon-γ in airway epithelial cells (14), a better understanding of these interactions and their functional consequences is of great interest.

Although EoE affects human subjects across the lifespan, pediatric patients more frequently display an inflammatory phenotype while a fibrostenotic phenotype is more common in adult patients (15–19). Studies investigating the natural history of EoE in human subjects indicate that likelihood of the fibrostenotic EoE phenotype increases with patient age and time to EoE diagnosis (15, 16). Delay in EoE diagnosis is also associated with increase in stricture in a time-dependent manner (20). Moreover, while gaps in clinical care of EoE patients are associated with progression to fibrostenosis (21), close follow-up is associated with a decrease in stricture in EoE patients (22). Taken together, these findings support the paradigm that chronic EoE inflammation promotes progression to fibrostenosis (15).

While aging is a risk factor for esophageal dysfunction and disease, our understanding of the biology underlying such associations is presently limited. In morphologically normal esophageal epithelium, aging has been associated with clonal expansion of cells expressing genetic alterations in cancer-associated genes, including *NOTCH1* and *TP53* (23, 24). Our recent study using transcriptomic profiling in murine esophageal epithelium further indicates that aging promotes mitochondrial dysfunction and predicts upregulation of stress response pathways, including oxidative stress response (25). These studies along with the inherent link between aging and disease duration in EoE patients led us to speculate that age-associated alterations in tissue biology may play a role in EoE fibrosis. Here, we employ experimental model systems to investigate how age specifically impacts EoE phenotype.

## Material and methods

### Animal experiments

All research for the current study complies with all relevant ethical regulations. All murine studies were performed in accordance with a protocol approved by the Temple University IACUC (Protocol Number: 5018). All animal experiments were conducted in accordance with institutional guidelines for animal research. All mice were maintained under controlled conditions with a 12 h light/dark cycle at an appropriate temperature and humidity. Young (<4 months old) and aged (≥18 months old) male and female C57BL/6 mice (Cat# 000664) were obtained from the Jackson Laboratory (USA). EoE-like inflammation was induced in young and aged mice using the previously described MC903 (calcipotriol)/OVA mouse model (26, 27) with minor modifications. All mice were sensitized by topical application of 20 μl of 10 μM MC903 (2700, Tocris, Bristol, UK) dissolved in 100% ethanol to both ears following ear scraping with a scalpel blade. MC903-only treated animals [MC903(+) OVA(−)] developed dermatitis at the site of MC903 application and served as vehicle controls. In experimental mice [MC903(+) OVA(+)], 10 μl at 10 mg/ml OVA (albumin from chicken egg white, A5503, Sigma-Aldrich, St. Louis, MO, USA) was applied to both ears directly following MC903 application. For the challenge phase of the protocol, experimental mice were subjected to oral gavage with 100 μl OVA (500 mg/ml in PBS) every other day from day 14 through day 32. Experimental mice also had *ad libitum* access to drinking water supplemented with OVA (15 g/ml) from day 14 through day 32. Vehicle controls were gavaged with an equal volume of PBS and provided *ad libitum* access to normal drinking water. At day 32 of the protocol, all animals were humanely euthanized then esophagi were dissected and processed for downstream analyses as described below.

### Primary cell isolation

All tissue isolation from mice was performed according to the approved IACUC protocol. Following dissection of whole esophagi, epithelium-enriched esophageal sheets were physically separated from underlying muscle using forceps. Epithelium-enriched esophageal sheets were then cut open longitudinally to expose the epithelial surface. Epithelium-enriched esophageal sheets were then incubated in 1 ml 1X Dispase (354235, Corning, NY, USA) in HBSS (14025-076, Gibco, Waltham, MA, USA) containing penicillin/streptomycin (1% v/v, 15140-122, Gibco, Waltham, MA, USA), gentamycin (5 μg/ml, Apex 25-533, Gibco, Waltham, MA, USA), and fungizone (500 μg/ml, 25-541, Genesee Scientific, San Diego, CA, USA) for 10 min at 37°C with shaking at 1,000 RPM (ThermoMixer F1.5 Eppendorf, St. Louis, MO, USA). Following removal from Dispase, epithelium-enriched esophageal sheets were chopped into 3 pieces with sharp scissors and incubated in 1 ml of 0.25% Trypsin-EDTA (25-510, Genesee Scientific, San Diego, CA, USA) for 10 min at 37°C with shaking at 1,000 RPM. Trypsin and tissue pieces were forced through a cell strainer (70 μm) into a 50 ml conical tube containing 4 ml 250 μg/ml soybean trypsin inhibitor (17975-029, Gibco, Waltham, MA, USA) in 1X PBS. Cells were pelleted at 1,000 RPM for 5 min then resuspended in 500 μl of complete “mouse” keratinocyte serum-free medium (KSFM; formulation described below). Cell number and viability were measured by automated cell count (Invitrogen Countess II FL) by mixing 10 μl of cell suspension with 10 μl 0.4% trypan blue solution (1:1) (T10282, Invitrogen, Waltham, MA, USA).

### Cell culture

Primary mouse esophageal epithelial cells (PMEC) were isolated from young or aged wild type C57/BL6 mice and passaged several times prior to use in experiments. PMECs were cultured in complete “mouse” KSFM that was generated by supplementing KSFM without calcium chloride (10725-018, Gibco, Waltham, MA, USA) with recombinant epidermal growth factor (1 ng/ml), bovine pituitary extract, (50 mg/ml), penicillin/streptomycin (1% v/v, 15140-122, Gibco, Waltham, MA, USA), and 0.018 mM CaCl_2_ (349610025, Acros Organics, Geel, Belgium) as previously described (25). PMECs were maintained in humidified atmosphere containing 5% CO_2_ at 37°C. Medium was changed every other day. 10 μM Y27632 (1254, Tocris, Bristol, UK) was added every time PMECs were split to limit cell death. Human fetal esophageal fibroblasts (FEF3; a generous gift from Anil K. Rustgi, MD; Columbia University) were cultured in DMEM media (10-013-CV, Corning, NY, USA) supplemented with 10% FBS (PS-NB1, Peak Serum, Wellington, CO, USA) and 1% penicillin/streptomycin.

### Fibroblast contraction assay

Effects of young and aged epithelium on fibroblast contraction were determined using fibroblast contraction assays as previously described (7, 13, 28). Briefly, 600,000 FEF3 cells with or without 300,000 young or aged PMECs were resuspended in 38 μl PBS and mixed with 462 μl media. Media consisted of 42 μl 10X EMEM (12-684F, Lonza, Basel, Switzerland), 47 μl FBS, 3.8 μl L-Glutamine (25-030-081, Gibco, Waltham, MA, USA), 9 μl sodium bicarbonate (17-613E, Lonza, Basel, Switzerland), 269.3 μl type I bovine collagen (5010; Advanced BioMatrix, Carlsbad, CA, USA), and 90 μl Matrigel basement membrane matrix (354234; Corning, NY, USA), which is composed of Laminin, Collagen IV, heparan sulfate proteoglycans, entactin/nidogen, and numerous growth factors. 0.5 ml of mix was seeded into each well of a 24-well plate and incubated at room temperature for 90 min to allow collagen polymerization. Gel was then mechanically detached from the plate wall and 0.5 ml DMEM (10% FBS, 1% *p*/s) was added to each well. Each plug was captured at 0.63X magnification using an Olympus MVX10 microscope (Olympus; Tokyo, Japan). The area of each plug was measured using ImageJ software (National Institutes of Health, USA) at 72 h. Relative plug area was obtained by measuring the final dimensions in comparison with its initial area. Relative plug area of 65% in wells with fibroblasts alone was set as the cut-off for a successful contraction assay. To test the effect of cytokines, 600,000 FEF3 cells were seeded as described above and treated with 500 ng/ml Periostin (29) (3548-F2; R&D Systems, Minneapolis, MN, USA) or 10 μg/ml E-Cadherin (30) (8505-EC; R&D Systems, Minneapolis, MN, USA) for 72 h. PBS was used as a vehicle control.

### Cytokine array

This experiment included equal numbers of male and female mice for 2 groups: young (*n* = 4) and aged (*n* = 4). Epithelium-enriched esophageal sheet from each mouse was mechanically homogenized on ice in 300 μl lysis buffer (EL-lysis buffer, Item J; RayBiotech, Peachtree Corners, GA, USA) with 1X Protease inhibitor Cocktail (AA-PI; RayBiotech, Peachtree Corners, GA, USA), then 250 μl of each sample with a protein concentration of 4 mg/ml were prepared for analysis. Mouse Cytokine Array Q4000 was performed by RayBiotech's ELISA Testing Service (Peachtree Corners, GA, USA). Lysates were diluted to 2 mg/ml. 200 cytokines/soluble factors were evaluated, 43 had 0% confidence level and were excluded. 42 were excluded as they were below the lower limit of detection (LLOD) for all samples and 1 was excluded as it was above the maximum limit of detection (MLOD) for all samples. We then set a threshold of cytokines/soluble factors with ≥ best confidence (percentage of samples between 3X LLOD and one third MLOD) level, resulting in exclusion of an additional 79 cytokines/soluble factors. This resulted in 78 cytokines/soluble factors with 75%–100% best confidence that were subjected to statistical testing.

### Histological analysis

Whole esophagi were dissected and fixed with 4% paraformaldehyde (#J19943-K2; Thermo Scientific, Waltham, MA, USA) or 10% Neutral Buffered formalin (5701; Epredia, Kalamazoo, MI, USA) for 12 h at 4°C. Tissues were washed with PBS then stored in 70% ethanol at 4°C prior to paraffin embedding. Hematoxylin and eosin (H&E) staining was performed at Fox Chase Cancer Center Histopathological Core (Philadelphia, USA). Masson's trichrome staining was performed according to the manufacturer's protocol (101987T, Richard-Allan Scientific, UK). Slides from each individual mouse experiment (two in total) were stained for H&E or Masson's trichrome 1 time. Slides were imaged using Leica DM1000 LED microscope (Leica, Germany). Lamina propria thickness was measured in 10–20 randomly selected fields of each Masson's trichrome stained tissue sample using Aperio ImageScope (Germany). Eosinophils were counted in lamina propria and epithelium across three high-power fields (HPFs; 400X magnification) per animal. Layers of murine esophagus were identified as delineated in Supplementary Figure S1.

### Statistical analysis

Two two-tailed *t*-test or two-way ANOVA followed by Tukey's multiple were used for statistical evaluation of data. *p* < 0.05 was used as the threshold for statistical significance. Statistical analysis was performed using GraphPad Prism (GraphPad Software, La Jolla, CA, USA).

## Results

### Advanced age is associated with fibrotic remodeling of the lamina propria in mice with EoE inflammation

**Figure 1:**
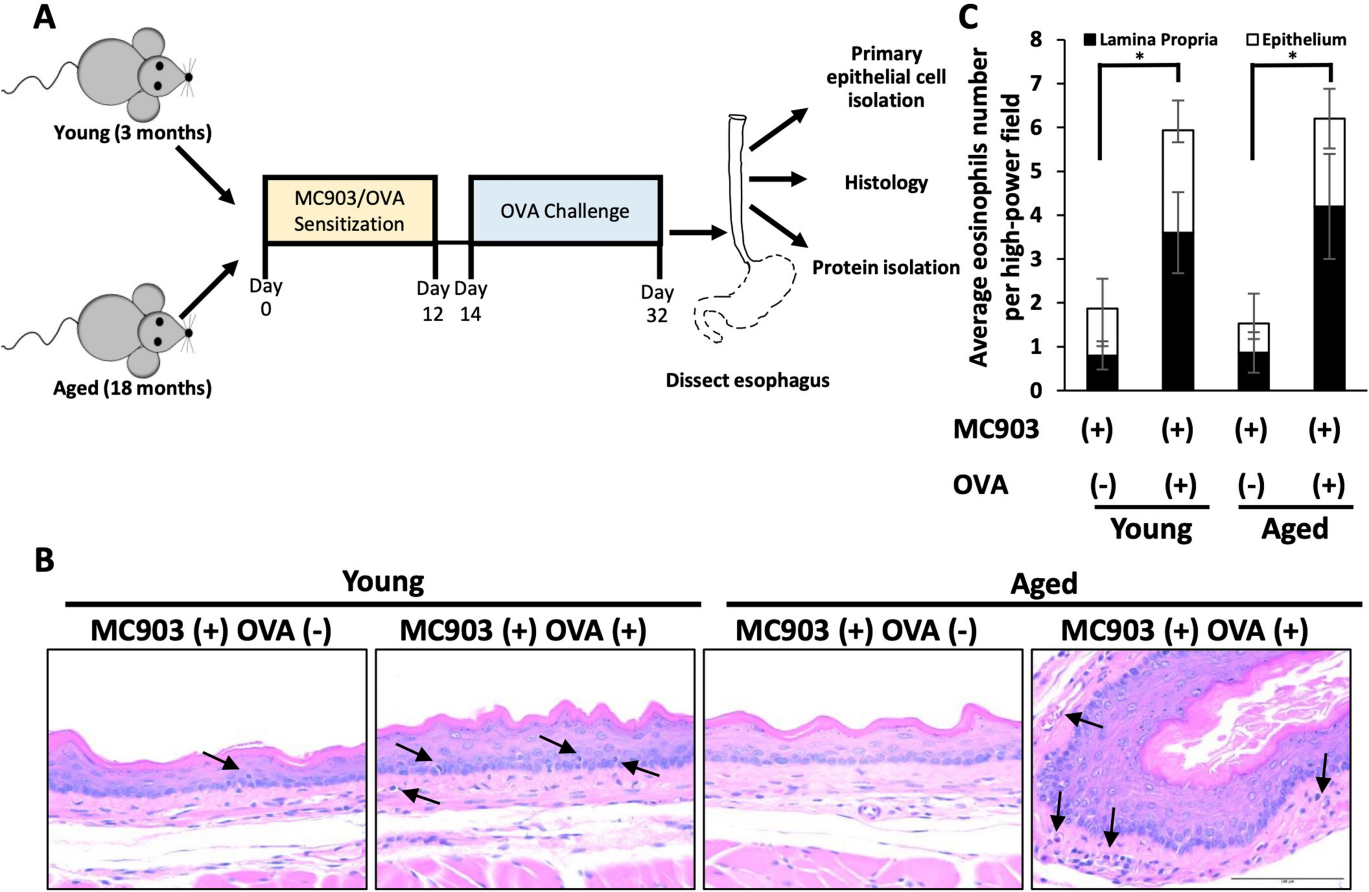
Impact of MC903/ovalbumin (OVA)-induced inflammation in young and aged mice. (**A**) Schematic of sensitization-challenge protocol for MC903-OVA-induced model of eosinophilic esophagitis. C57BL/6 mice were sensitized for 12 days with MC903 ± OVA *via* ear scratch to induce development of atopic dermatitis–like skin lesion. From days 14–32, OVA-treated mice were challenged via intragastric OVA every other day with continuous access to OVA-supplemented drinking water. (**B**) Representative H&E-stained sections from mice treated with MC903 ± OVA. Arrows indicate eosinophils. Scale bar, 100 μm. Data are from two independent experiments including four groups: young MC903(+) OVA(−) (*n* = 5); young MC903(+) OVA(+) (*n* = 6); aged MC903(+) OVA(−) (*n* = 6); and aged MC903(+) OVA(+) (*n* = 6). (**C**) Eosinophils were counted in lamina propria and epithelium across three high-power fields (HPF; 400X magnification) per animal. Bar graph represents average per experimental group (mean ± SEM). Two-way ANOVA testing with Tukey's correction was used to determine significance. *p ≤ 0.05.

**Figure 2:**
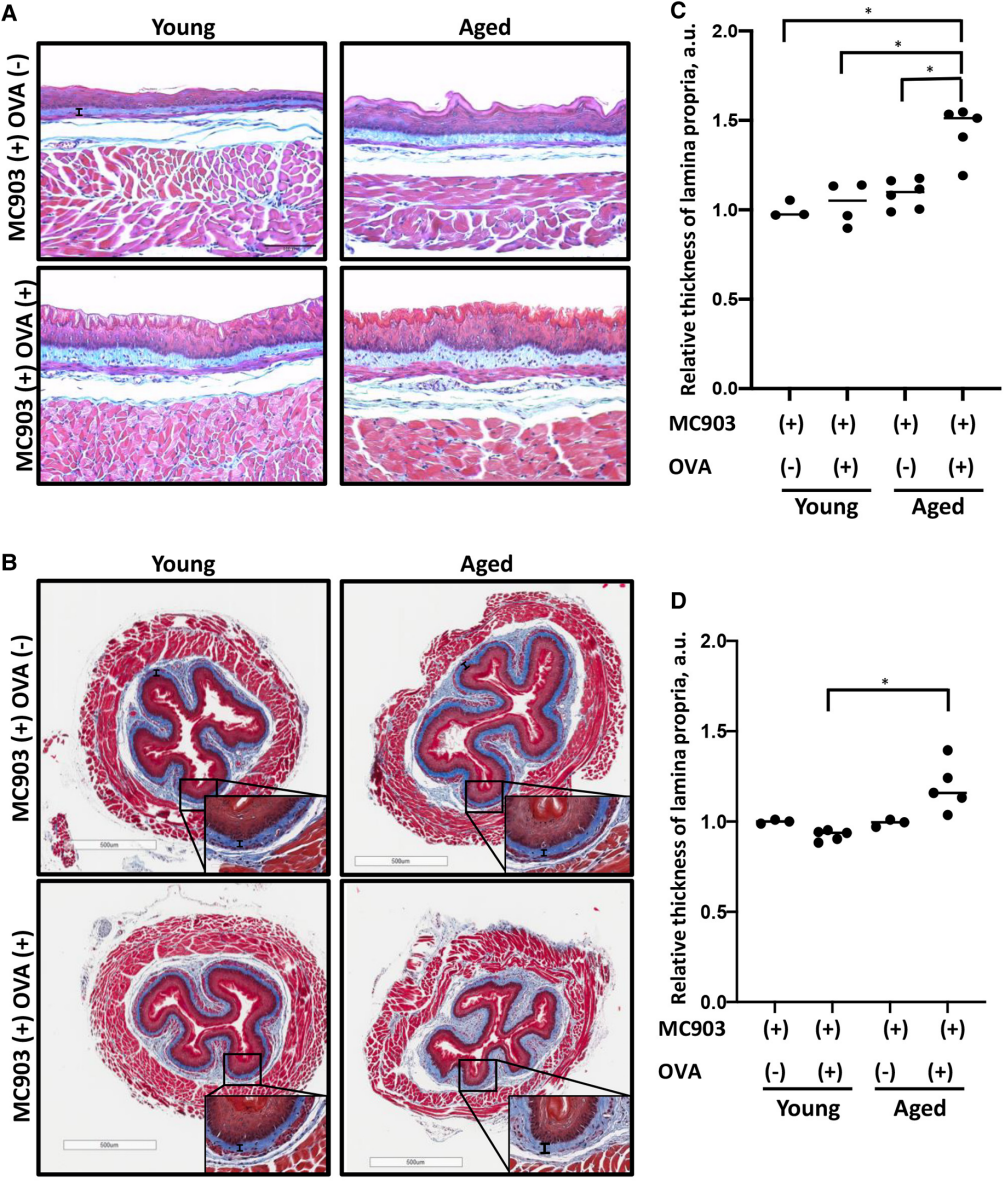
Lamina propria fibrosis is increased in aged mice with MC903/ovalbumin (OVA)-induced EoE. Esophageal tissue sections from young and aged mice treated with MC903 ± OVA were subjected to trichrome staining to assess fibrosis. (**A,B**) Representative sections of esophagi cut in the sagittal (in **A**) or traverse plane (in **B**). Scale bar, 100 μm (in **A**), 500 μm (in **B**). Thickness of lamina propria is delineated by vertical black bar. Insets (in **B**) were imaged at 400X magnification. (**C,D**) Lamina propria thickness relative to that of MC903(+) OVA(−)-treated young animals was determined for sections of esophagi cut in the sagittal (in **C**) or traverse (in **D**) plane. 11-20 individual measurements were taken for each animal, where each dot represents mean for one animal and bar represents median. Two-way ANOVA testing with Tukey's correction was used to determine significance. *p ≤ 0.01. Data are from two independent experiments including four groups: young MC903(+) OVA(−) (*n* = 3); young MC903(+) OVA(+) (*n* = 4 and 5, respectively); aged MC903(+) OVA(−) (*n* = 6 and 3, respectively); and aged MC903(+) OVA(+) (*n* = 5).

To investigate the impact of aging upon fibrotic remodeling in EoE, we subjected young (<4 months) and aged (≥18 months) mice to MC903/OVA sensitization and challenge over the course of 32 days (Figure 1A). With this protocol, increased eosinophilia was detected in esophageal mucosa of experimental mice treated with MC903 and OVA [MC903(+) OVA (+)] as compared to vehicle controls treated with MC903 alone [MC903(+) OVA (−)] and these results were similar in young and aged mice (Figures 1B,C). By contrast, expansion of the lamina propria was uniquely apparent in aged experimental MC903(+) OVA (+) mice (Figures 2A,B). Masson's trichrome staining further revealed marked collagen deposition in aged experimental MC903(+) OVA (+) mice (Figures 2A,B). Indeed, when compared to young experimental MC903(+) OVA (+), a significant increase in lamina propria thickness was found in aged experimental MC903(+) OVA (+) mice (Figures 2C,D). This finding was apparent in tissue sections from esophagi cut in the sagittal and traverse planes (Figures 2A–D) and suggests that age-associated alterations in esophageal tissue biology may contribute to fibrosis in EoE.

### Young esophageal epithelial cells more efficiently limit fibroblast contraction as compared to aged esophageal epithelial cells

**Figure 3:**
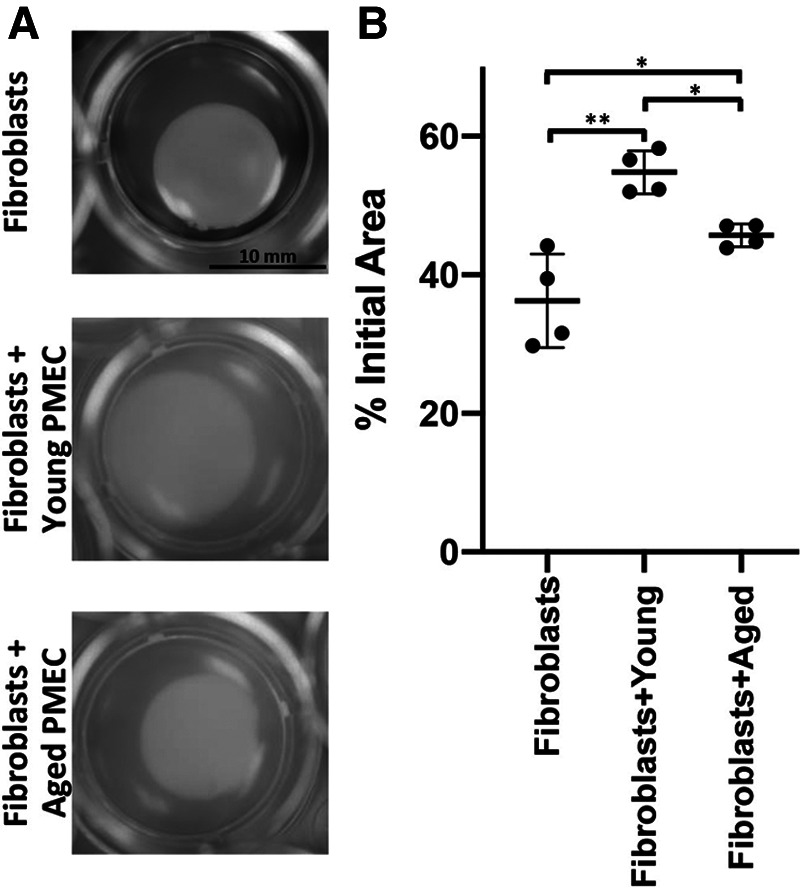
Impact of esophageal epithelial cells from young and aged mice upon fibroblast contraction. Primary murine esophageal epithelial cells (PMECs) were isolated from young or aged wild type C57/BL6 mice and passaged several times prior to experiment. Fetal esophageal fibroblast cell line FEF3 was co-cultured with young or aged PMECs in collagen plug contraction assay for 72 h. (**A**) Representative images for contraction of collagen plugs following 72 h of fibroblasts alone and co-cultured with aged or young PMECs. Images were captured at 72 h using an Olympus MVX10 microscope (0.63X objective). (**B**) Quantitative analysis of fibroblast-mediated gel contraction. Relative plug area was obtained by measuring final dimensions in comparison with initial area. Each dot represents mean for one of four independent experiments performed in triplicate and bar represents mean ± SD. Two-way ANOVA testing with Tukey's correction was used to determine significance. *p ≤ 0.05; **p ≤ 0.001.

As epithelial-stromal crosstalk has been shown to influence fibroblast behavior in the context of EoE (31), we next aimed to determine if esophageal epithelial cell age has an impact upon fibroblast contractility. We first generated PMECs (primary murine esophageal epithelial cells) from wild type young and aged mice. PMECs were subsequently embedded in a collagen plug along with FEF3 esophageal fibroblasts. Area of the plug then was measured after 72 h of co-culture as a read-out for fibroblast activity. These assays revealed that while both primary esophageal epithelial cells from young mice limited fibroblast-mediated matrix contraction, this inhibition was more robust with PMECs from young mice as compared to those from aged mice (Figures 3A,B). These findings support the premise that esophageal epithelial cells provide anti-fibrotic cues that become dampened in the context of aging.

### Exploration of soluble factors that may contribute to age-associated fibrosis

**Figure 4:**
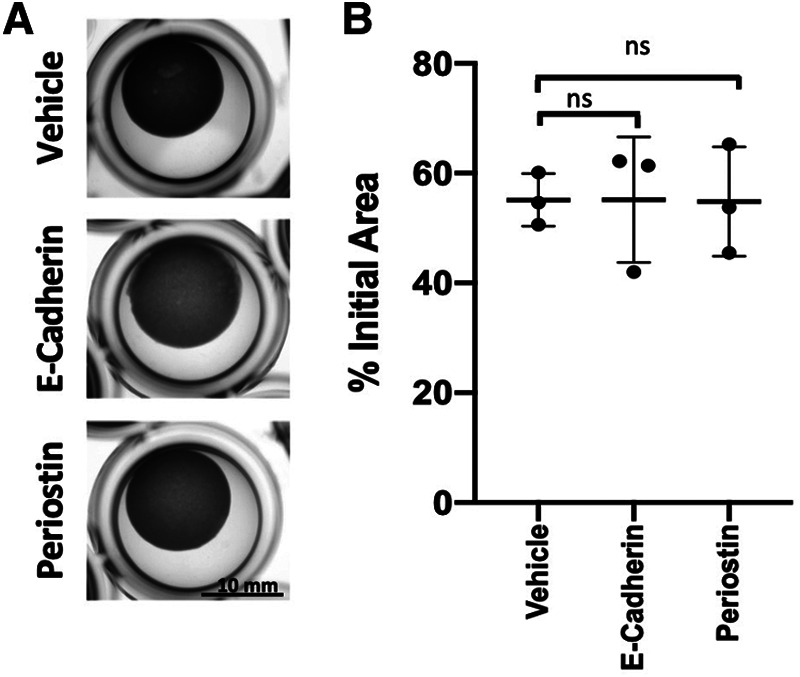
Impact of Periostin and soluble E-cadherin upon fibroblast contraction. (**A**) Representative images of FEF3-embedded collagen plugs following treatment with vehicle (PBS), E-Cadherin (10 μg/ml) or Periostin (500 ng/ml) for 72 h. (**B**) Quantitative analysis of fibroblast-mediated gel contraction. Relative plug area was obtained by measuring final dimensions in comparison with initial area. Each dot represents mean for one independent experiment performed in triplicate and bar represents mean ± SD. Statistical analysis was performed using two-way ANOVA (ns, not significant).

**Figure 5:**
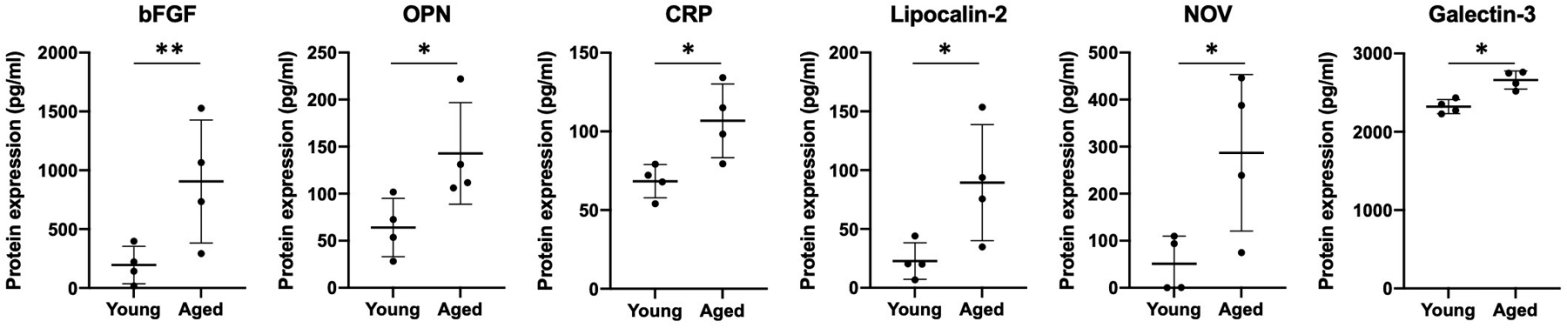
Cytokines displaying age-associated differential expression in murine epithelium-enriched tissue sheets. Dot plots display level of basic fibroblast growth factor (bFGF), Osteopontin (OPN), C-reactive protein (CRP), Lipocalin-2, Nephroblastoma overexpressed (NOV/CCN3) and Galectin-3 expression in epithelium-enriched tissue fraction from young or aged mice. Cytokine/soluble factor values were determined by RayBiotech Mouse Cytokine Array Q4000 with 75%–100% of best confidence. Data are from an experiment including two groups: young (*n* = 4) and aged (*n* = 4) and presented as mean ± SD. Statistical analysis was performed using two-tailed *t*-test (**p value < 0.005; *p value < 0.05).

The pro-fibrotic matricellular protein Periostin has been linked to EoE (32, 33) and age-associated increase in Periostin expression has been linked with cardiac fibrosis (34). As such, we aimed to determine if addition of exogenous Periostin may influence fibroblast contraction. At a concentration of 500 ng/ml, Periostin promotes expression of TGF-β and Collagen I in lung fibroblasts (29); however, this concentration failed to promote contraction of esophageal FEF3 fibroblasts (Figures 4A,B). Soluble E-Cadherin fragments may be generated during EMT (35, 36), a process that contributes to EoE fibrosis (10, 11, 13) and has been linked to age-associated fibrosis in multiple organs (37). Although 10 μg/ml soluble E-Cadherin cadherin (N-terminal domain that is soluble upon protease cleavage) has been demonstrated to activate epidermal growth factor receptor signaling in kidney cells (30), this concentration was insufficient to promote contraction of esophageal FEF3 fibroblasts (Figures 4A,B) In order to more broadly explore soluble factors produced by aged esophageal epithelium, we performed an ELISA-based mouse cytokine array in epithelium-enriched esophageal sheets from young and aged mice (Supplementary File S1). Of the 200 cytokines/soluble factors whose expression was evaluated, 78 were detectable with 75%–100% of best confidence across all samples from young and aged mice (Table 1). Among these 78 targets, levels of bFGF (basic fibroblast growth factor), OPN (Osteopontin), CRP (C-reactive protein), Lipocalin-2, NOV (Nephroblastoma overexpressed; CCN3) and Galectin-3 were significantly upregulated in aged mice compared to their young counterparts (Figure 5). This cytokine array data is hypothesis-generating, identifying several cytokines/soluble factors that may play a role in age-associated EoE fibrosis.

**Table 1:** Top 25 differently expressed cytokines/soluble factors ranked by *p* value.

Cytokine/Soluble factor	Average expression (pg/ml; mean ± SD)		*p*	LLOD	MLOD
	Young (*n* = 4)	Aged (*n* = 4)			
Galectin-3	2,321 ± 90	2,662 ± 115	0.003**	4.0	2,000
CRP	68 ± 11	107 ± 23	0.024*	0.9	741
NOV	51 ± 59	287 ± 166	0.037*	11.4	7,407
bFGF	196 ± 159	905 ± 522	0.041*	7.7	5,000
Lipocalin-2	23 ± 16	89 ± 49	0.042*	1.4	6,173
OPN	64 ± 31	143 ± 54	0.045*	9.7	11,111
Chordin	368 ± 144	135 ± 162	0.075	20.0	10,000
PlGF-2	2 ± 2	4 ± 2	0.078	0.3	185
GITR	108 ± 46	53 ± 30	0.091	8.0	4,000
CD36	1,059 ± 447	2,653 ± 1,536	0.093	34.8	1,11,111
Decorin	7,496 ± 640	8,124 ± 152	0.105	1.1	5,000
Fractalkine	22 ± 4	26 ± 3	0.108	0.9	6,173
TWEAK R	207 ± 124	916 ± 775	0.121	28.8	25,000
IL-1α	36 ± 21	63 ± 23	0.123	0.2	123
Nope	205 ± 87	337 ± 122	0.129	17.5	10,000
IGF-1	26 ± 12	43 ± 17	0.148	0.2	617
P-selectin	1,462 ± 377	2,018 ± 602	0.168	8.0	4,000
Fcy RIIB	76 ± 26	110 ± 36	0.172	0.8	5,556
IL-3 Rβ	1,108 ± 1,204	2,604 ± 1,685	0.198	80.0	40,000
Adiponectin	11,793 ± 3,467	15,394 ± 3,582	0.199	20.0	10,000
6Ckine (CCL21)	794 ± 800	1,832 ± 1,284	0.219	40.0	20,000
CD48	173 ± 79	272 ± 123	0.225	4.0	2,000
Eotaxin	153 ± 63	205 ± 55	0.265	1.0	1,000
IL-33	31 ± 10	55 ± 39	0.272	4.5	4,000
Neprilysin	564 ± 291	936 ± 558	0.282	24.7	11,111

LLOD, lower limit of detection; MLOD, maximum level of detection; SD, standard deviation.

* *p* values determined by *t*-test denoting *p* < 0.05.

** *p* values determined by *t*-test denoting *p* < 0.005.

## Discussion

In the current study, we leveraged an allergen-induced murine EoE model to test how age specifically impacts EoE phenotype. By exposing young and aged animals to MC903/OVA-induced EoE inflammation for an identical exposure period, we found that evidence of fibrosis is uniquely present in aged mice, indicating that age-associated tissue biology alterations contribute to EoE fibrosis. Natural history studies in human subjects support the notion that chronic inflammation promotes disease progression to fibrostenosis (15, 16, 20–22). Findings from the current study are not in conflict with this hypothesis, but rather complement it. In human subjects, EoE inflammation may be present over years or even decades, and during this time period the inflammatory milieu can cooperate with age-associated alterations in tissue biology to drive fibrosis.

A key question that remains is: *What are the age-associated alterations in tissue biology that contribute to EoE fibrosis?* Here, we demonstrate that while both young and aged epithelial cells limit fibroblast contraction in *ex vivo* co-culture experiments, aged epithelial cells do so less effectively as compared to their young counterparts. To define potential mediators of this phenotype, we focused upon soluble factors. Although Periostin has been linked with fibrosis in EoE (32, 33) and age-associated fibrosis in other tissues (34), exogenous Periostin failed to promote FEF3 contractility. Likewise, although soluble E-Cadherin has been shown to be increased in cells undergoing EMT (35, 36) and EMT has been associated with both EoE- and age-associated fibrosis (10, 11, 13, 37), addition of soluble E-Cadherin to culture medium in FEF3 fibroblast contraction assays did not effect contractility. While dose response experiments are necessary to more thoroughly examine these factors as mediators of age-associated fibrosis, neither Periostin nor soluble E-Cadherin was found to show significant differential expression in our cytokine array experiments comparing expression of 200 soluble factors in esophageal epithelium-enriched tissue fractions in young and aged mice (Supplementary File S1). This cytokine array did, however, identify 6 cytokines/soluble factors whose expression was significantly upregulated in epithelium-enriched esophageal tissue of aged mice: bFGF, OPN, CRP, Lipocalin-2, NOV and Galectin-3. Among these, bFGF, OPN, and Galectin-3 have been shown to be upregulated in biospecimens from EoE patients with bFGF detected in plasma, OPN in urine, and Galectin-3 in esophageal biopsies (38–40). Although these factors have not been linked to EoE fibrosis, they have been associated with fibrosis in other tissue sites. The heparin-binding growth factor bFGF is a potent inducer of fibroblast proliferation that promotes fibrosis in the lung (41). OPN, a matricellular protein, and Galectin-3, a carbohydrate-binding protein, have both been positively associated with fibrosis in various organs, including the heart, liver, kidney, and lung through mechanisms that include stimulation of fibroblast proliferation and collagen production (42–48). The glycoprotein Lipocalin-2 (also known as neutrophil gelatinase-associated lipocalin), the matricellular protein NOV3 (also known as cellular communication factor 3), and the marker of inflammation CRP have yet to be linked to EoE, although they have each been associated with fibrosis with NOV3 specifically identified as an inhibitor of fibrosis in the kidney (49–52). Future investigations will examine the impact of addition of exogenous bFGF, OPN, CRP, Lipocalin-2, NOV and Galectin-3 upon FEF3 esophageal fibroblast contractility.

Esophageal epithelium may also influence fibroblast behavior through mechanisms that are not dependent upon soluble factors. Given that the co-culture system used herein to examine the effect of epithelial cell age upon fibroblast contractility facilitates epithelial-fibroblast contact, it is possible that such direct contact generates signals to promote fibrosis. This could be evaluated by determining the impact of conditioned media from young and aged esophageal epithelial cells on fibroblast contractility. Our published work indicates that aged esophageal epithelium exhibits mitochondrial dysfunction, including decreased activity of Complex I of the electron transport chain, along with evidence of increased oxidative stress. As altered mitochondrial bioenergetics and reactive oxygen species have been implicated in idiopathic pulmonary fibrosis (53), studies investigating these factors in age-associated EoE fibrosis may prove fruitful. Moreover, accumulation of somatic mutations in genes associated with esophageal cancer occurs with age in physiologically normal esophageal epithelium, raising the possibility that genetic events may alter esophageal epithelial biology to promote age-associated EoE fibrosis. Age-associated alterations in fibroblasts may also play a role in EoE-associated fibrosis. Indeed, fibroblasts from non-EoE adult subjects show a baseline contractility that is greater than that seen in fibroblasts derived from their pediatric counterparts (7). Adult fibroblasts also fail to exhibit a response to TGF-β whereas pediatric fibroblasts show increased contractility in response to this cytokine (7).

As we do not detect fibrosis in aged mice in the absence of EoE inflammation, it is important to consider how EoE inflammation interacts with age-associated changes in tissue biology. Beyond cytokine/soluble factor alterations occurring in the esophagus solely as a result of age, future studies may include examination of the effect of MC903/OVA-induced inflammation on cytokine/soluble factor levels in epithelium-enriched esophageal sheets from young and aged mice. Additionally, inflammatory cells that have infiltrated epithelium-enriched esophageal sheets play an important role in EoE pathobiology. Indeed, soluble factors from inflammatory cells, including eosinophil-generated TGF-β and IL-1β (8), and endothelial cell-derived TSPAN12 (54) have been implicated in EoE fibrosis. TGF-β has also been demonstrated to promote induction of effector T cells but not regulatory T cells in the context of allergic airway inflammation (55), highlighting the importance of crosstalk with the immune system in allergic inflammation. EoE inflammation may also impact molecular tissue age as suggested by a recent study that reported evidence of epigenetic events associated with premature molecular aging in esophageal biopsies from EoE patients but not those from normal controls (56). This finding is intriguing as it may have implications for determination of whether an EoE patient will develop fibrostenotic disease. As fibrosis is detected in a subset of children with EoE (57) and fibrostenosis is not found in all adult EoE patients even in the context of untreated disease and long-term inflammation (20), it is clear that chronological age is not always directly linked to EoE fibrosis. It is tempting to speculate, however, that epigenetic modulation of tissue aging plays a role in determination of whether an EoE patient will develop fibrostenotic disease.

**Figure 6:**
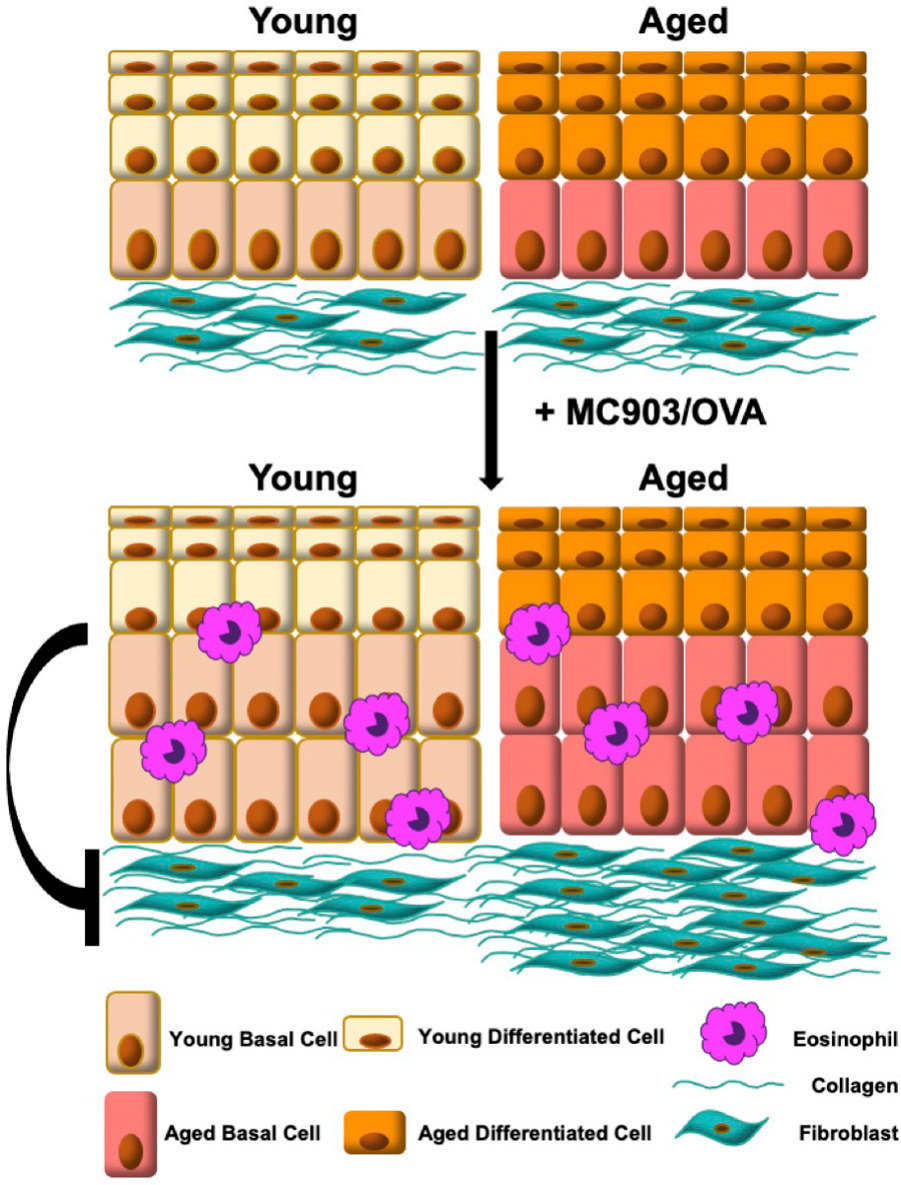
Model: signals from young esophageal epithelium limit fibroblast contraction and are diminished upon tissue aging, promoting fibrosis in the presence of eosinophilic esophagitis inflammation.

Taken together, findings from the current study reveal a previously unappreciated role for age-associated changes in tissue biology, specifically that of esophageal epithelium, in EoE fibrosis. We propose that epithelial cells act to suppress fibroblast contractility and that this protective suppression is diminished with age, promoting fibrosis in the presence of EoE inflammation (Figure 6). Mechanistic evaluations of soluble factors showing differential expression in aged epithelium-enriched esophageal sheets suggest that E-cadherin and Periostin are not likely mediators of age-associated EoE fibrosis. Other targets showing differential expression in epithelium-enriched esophageal sheets from young and aged mice further provide hypothesis-generating data that may be explored to define novel pro-fibrotic mechanisms in the esophagus. Such studies are critical as understanding the signals that regulate EoE fibrosis holds the promise of identifying targets that will facilitate early detection, prevention, and therapy of fibrotic lesions in EoE patients.

## Data Availability

The authors declare that all data supporting the findings of this study are available within the article and its supplementary information files or from the corresponding author upon reasonable request.
