# Supplementary material for: A role for age-associated alterations in esophageal epithelium in eosinophilic esophagitis-associated fibrosis
Source: Front Allergy. 2022 Dec 15;3:983412. doi: 10.3389/falgy.2022.983412 (PMC9798296; doi:10.3389/falgy.2022.983412)
Supplement: Supplementary file 1 [file Datasheet1.pdf]

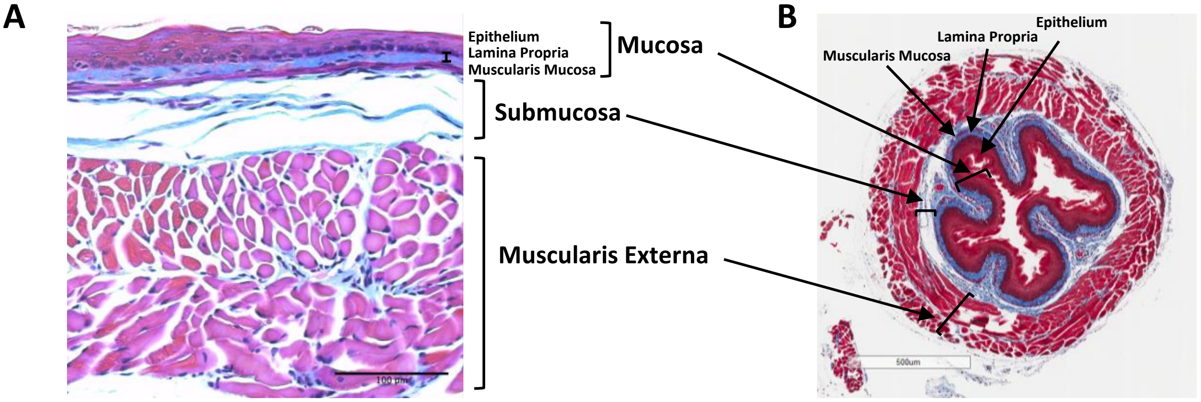

Supplementary Figure S1. Tissue layers of the murine esophagus. (**A, B**) Representative sections of esophagi cut in the sagittal (in **A**) or traverse plane (in **B**) stained for trichrome. Scale bar, 100 µm (in **A**), 500 µm (in **B**). Lamina propria is delineated by vertical black bar (in **B**).
